# Exposure to viral and bacterial pathogens among Soay sheep (*Ovis aries*) of the St Kilda archipelago

**DOI:** 10.1017/S0950268816000017

**Published:** 2016-02-01

**Authors:** A. L. GRAHAM, D. H. NUSSEY, J. O. LLOYD-SMITH, D. LONGBOTTOM, M. MALEY, J. M. PEMBERTON, J. G. PILKINGTON, K. C. PRAGER, L. SMITH, K. A. WATT, K. WILSON, T. N. McNEILLY, F. BRÜLISAUER

**Affiliations:** 1 Department of Ecology & Evolutionary Biology, Princeton University, Princeton, NJ, USA; 2 Fogarty International Center, National Institutes of Health, Bethesda MD, USA; 3 Institute of Evolutionary Biology, School of Biological Sciences, University of Edinburgh, Edinburgh, UK; 4 Department of Ecology & Evolutionary Biology, University of California Los Angeles, Los Angeles, CA, USA; 5 Moredun Research Institute, Pentlands Science Park, Penicuik, Midlothian, UK; 6 Animal and Plant Health Agency, New Haw, Addlestone, Surrey, UK; 7 Lancaster Environment Centre, Lancaster University, Lancaster, UK; 8 SAC Consulting Veterinary Services, Scotland's Rural College, Inverness, UK

**Keywords:** Epidemiology, parasites

## Abstract

We assessed evidence of exposure to viruses and bacteria in an unmanaged and long-isolated population of Soay sheep (*Ovis aries*) inhabiting Hirta, in the St Kilda archipelago, 65 km west of Benbecula in the Outer Hebrides of Scotland. The sheep harbour many metazoan and protozoan parasites but their exposure to viral and bacterial pathogens is unknown. We tested for herpes viral DNA in leucocytes and found that 21 of 42 tested sheep were infected with ovine herpesvirus 2 (OHV-2). We also tested 750 plasma samples collected between 1997 and 2010 for evidence of exposure to seven other viral and bacterial agents common in domestic Scottish sheep. We found evidence of exposure to *Leptospira* spp., with overall seroprevalence of 6·5%. However, serological evidence indicated that the population had not been exposed to border disease, parainfluenza, maedi-visna, or orf viruses, nor to *Chlamydia abortus*. Some sheep tested positive for antibodies against *Mycobacterium avium* subsp. *paratuberculosis* (MAP) but, in the absence of retrospective faecal samples, the presence of this infection could not be confirmed. The roles of importation, the pathogen–host interaction, nematode co-infection and local transmission warrant future investigation, to elucidate the transmission ecology and fitness effects of the few viral and bacterial pathogens on Hirta.

## INTRODUCTION

The species composition of an island community is expected to be shaped by both the distance of the island from the mainland (e.g. as a predictor of immigration rates) and by the area of the island (e.g. as a predictor of extinction rates) [1]. Parasites and pathogens, too, must immigrate and avoid extinction in order to prosper on islands. Indeed, the establishment of infectious diseases on remote islands is probably hindered by low immigration rates (especially if founder hosts arrive uninfected) and by low or variable host densities [2]. Small host populations are a particular obstacle for acute immunizing infections, for which a positive relationship between population size and pathogen persistence is expected [3] (the classic example being measles [4]). Accordingly, depauperate parasite communities have been reported on several islands compared to mainland populations [5].

**Fig. 1.**
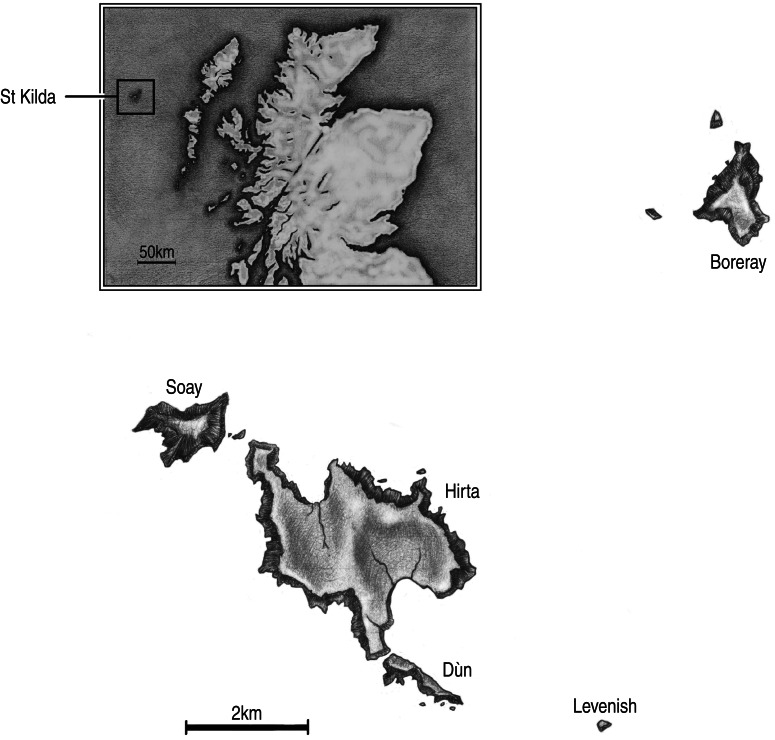
Map of the St Kilda archipelago, with islands including Hirta, where the focal population of sheep live. Location of the archipelago relative to mainland Scotland is shown in the inset. (The maps were drawn by Rebecca Holland.)

Soay sheep (*Ovis aries*) inhabiting the island of Hirta, in the St Kilda archipelago of the Outer Hebrides of Scotland (Fig. 1), present an opportunity to study the role of parasites and pathogens in the life histories of island ruminants. Since Neolithic times, the sheep have lived on St Kilda, isolated from other Hebridean islands by ~65 km and from dense sheep populations on the Scottish mainland by a further ~60 km. Historically, visitor traffic was minimal but it is currently rising. A few dozen people live on Hirta year-round, on a shift-work basis with trips to the mainland between shifts. The focal sheep population has been unmanaged since 1930, when 103 founders were moved to Hirta from Soay [6]. The population undergoes boom-bust dynamics, with overwinter die-offs caused by forage limitation, harsh winter weather, and parasites [7]. Known infectious agents of the sheep include an array of metazoan and protozoan parasites [8], including strongyle nematodes [9], coccidia [10], and cryptosporidia [11]. Other than the potential for transmission of trypanosomes on Hirta by ectoparasitic keds [12] which have been eliminated from domestic sheep, this represents a depauperate parasite fauna compared to sheep on the Scottish mainland (e.g. notably lacking the flatworm *Fasciola hepatica*) [8].

These parasites, especially the nematodes, are thought to be the most important natural enemies faced by the sheep, given the dearth of predators on Hirta. However, the role, if any, of viruses and bacteria in the ecology of this island population is unknown. Here, we report results of tests for exposure of the sheep to eight viral and bacterial infectious agents common in sheep elsewhere in Scotland. For example, in a randomized serosurvey comprising 125 Scottish flocks in 2007, 32·8% of flocks tested positive for previous exposure to border disease virus (BDV) and 96·0% to parainfluenza-3 virus (PI3 V) [13], both of which mainly cause transient infections followed by robust immunity. Furthermore, we were interested in common ovine pathogens that cause chronic infections. These included ovine herpesvirus (OHV-2), maedi-visna virus (MVV) and *Chlamydia abortus*, the most common infectious abortifacient in the UK [14]. Finally, we were interested in *Mycobacterium avium* subsp. *paratuberculosis* (MAP) and *Leptospira* spp., both of which infect a wide range of species, induce chronic carriage, and may survive in the environment for extended periods of time. Our results confirm epidemiological isolation of the Soay sheep but also suggest potential for local transmission of herpesvirus and leptospire infections, in addition to the highly prevalent nematodes and coccidia [9, 10].

## METHODS

The sheep in the Village Bay area of the 6·3 km^2^ island of Hirta in the St Kilda archipelago (57° 49′ N/08° 35′ W) have been the subject of longitudinal, individual-based demographic, genetic and phenotypic study since 1985 [6]. Each April, ~95% of lambs are tagged within a week of birth. The population is thus characterized by birth pulses, the timing of which varies modestly among years (e.g. median birth date ranged from 15 April to 25 April for lambs born between 1986 and 2000) and may reflect density-dependent demographic rates [6]. Each August, ~50% of the population is re-captured, and blood samples are collected into heparin, stored at 4 °C and, within 24 h of collection, centrifuged at ~1500 ***g*** for 15 min. Plasma and cellular fractions are separated and stored at −20 °C.

### Serosurvey for evidence of exposure to seven viral and bacterial infectious agents

For our primary serosurvey across seven pathogens and 14 years, we selected 750 plasma samples for testing, using a random number generator within each age group, capture year and sex. These 750 demographically representative but otherwise randomly selected samples came from 659 individual sheep captured each August during the years 1997–2010 (Table 1) and included samples from 350 yearlings and 400 adults (aged 3–5 years), with 91 individuals sampled as both yearling and adult. We tested males and females equally. In pilot serosurveys across fewer pathogens and sampling years, we had previously commissioned tests for MVV in 196 plasma samples collected in 1986, and for MVV, BDV, *C. abortus* and MAP in a separate set of 50 plasma samples collected in 2000. For each pilot and for the primary serosurvey, we distributed aliquots to governmental and commercial agencies specializing in the tests required (Table 2, with citations to detailed descriptions of all serological methods): the SAC Consulting Veterinary Services (Disease Surveillance Centres in Inverness and St Boswells), the Animal and Plant Health Agency (APHA, a division of the Department for Environment, Food and Rural Affairs; Defra), and the Moredun Research Institute (MRI).

**Table 1.** Number of sheep tested in our major serosurvey, according to age group (yearlings vs. adults, 3–5 years of age) and capture year

Sampling year	Yearlings tested	Adults tested
1997	0	54
1998	0	23
1999	0	40
2000	42	23
2001	42	19
2002	0	15
2003	42	28
2004	42	32
2005	0	8
2006	22	26
2007	31	49
2008	43	19
2009	43	22
2010	43	42
Total	350	400

Of the 350 yearlings tested, 91 were also tested as adults. We thus tested 750 plasma samples collected from 659 individual animals. Samples from yearlings were available from each August of years 2000–2010, except for 2002 and 2005 due to near-complete mortality of that age group over the preceding winter. Samples from adult sheep were available annually from 1997 to 2010, but with fewer samples collected following the large population die-offs (e.g. in 2002 and 2005).

**Table 2.** *(*a*)* Viral and (b) bacterial infectious agents for which samples were tested, and number and percentage positive for these agents, across all sheep and years tested in our major survey

Infectious agent	Taxon, tissue affected	Method, tester, ref.	No. positive/ no. tested (%)
(***a*) Viruses**			
Border disease virus (BDV)	Pestivirus of intestinal and urogenital tracts	ELISA [32], MRI	0/400 (0%)
Parainfluenza-3 virus (PI3 V)	Paramyxovirus of respiratory tract	ELISA [33], MRI	0/400 (0%)
Orf virus	Parapoxvirus of skin	ELISA, MRI	0/400 (0%)
Maedi-visna virus (MVV)	Lentivirus of immune and respiratory systems	ELISA [16], AGID [24], SACCVS*	0/659 (0%)
Ovine herpes virus-2 (OHV-2)	Herpesvirus affecting lymphocytes	PCR [21], MRI	21/42 (50%)
**(*b*) Bacteria**			
Agent of ovine chlamydial abortion	*Chlamydia abortus,* uterine and placental epithelium	CFT [16], ELISA [27], SACCVS, MRI*	0/659 (0%)
Agent of Johne's disease	*Mycobacterium avium* subsp. *paratuberculosis,* intestine	ELISA [16], SACCVS	8†/659 (1·2%†)
Agent of leptospirosis	*Leptospira* spp., primarily urogenital tract	MAT [16], APHA	43/659 (6·5%)

The table includes the taxonomic identity of each infectious agent, tissues affected, detection methods [enzyme-linked immunosorbent assay (ELISA); agar gel immuno-diffusion (AGID); polymerase chain reaction (PCR); complement fixation test (CFT); or microscopic agglutination test (MAT)] and testing agencies [Moredun Research Institute (MRI); SAC Consulting Veterinary Services (SACCVS) Disease Surveillance Centres (DSC) at Inverness and St Boswells; or Animal and Plant Health Agency (APHA)]. The final column shows results in terms of the number (and percentage) of individual sheep positive out of the number tested.

* Denotes two ELISA and 32 CFT positive samples that were then determined to be false positives by AGID for MVV [24] and POMP-specific ELISA for *C. abortus* [27].

† Denotes ELISA positives but we could not undertake MAP bacterial culture to confirm whether these were true positives.

Where possible, positive results by initial screening were followed with appropriate tests of heightened specificity. Our most detailed follow-up was for *Leptospira* spp. For the 43 individuals positive by generic microscopic agglutination test (MAT) at a plasma dilution of ⩾1:100, we conducted a follow-up MAT to quantify antibodies capable of agglutinating bacteria from five candidate serovars across three species: *Leptospira interrogans* serovar (sv.) Hardjo and *L. interrogans* sv. Pomona; *L. borgpetersenii* sv. Hardjo and *L. borgpetersenii* sv. Ballum; and *L. biflexa* sv. Patoc. We selected these because they could plausibly be transmitted on Hirta: the first three can be maintained within ruminant populations [15], Ballum is maintained in rodent reservoirs [16] such as the endemic Hirta mouse, and Patoc is a saprophyte [17]. We note that *L. interrogans* sv. Hardjo and *L. borgpetersenii* sv. Hardjo are the most prevalent in sheep on the Scottish mainland [18]; antibodies against them have been detected in 6·3% of domestic sheep across England and Wales [15]. Serovar-specific MAT requires live *Leptospira* cultures in liquid medium. Starting with 1:50 dilution, we mixed a series of twofold dilutions of plasma (1:100, 1:200, etc.) with equal volumes of culture. After incubation at 30 °C, we read results by dark-field microscopy. The last dilution at which >50% of bacteria remained agglutinated was reported as the endpoint titre [19]. We applied positivity cut-offs of 1:100 for screening and 1:50 for sensitive confirmatory testing [16].

### Polymerase chain reaction (PCR) of blood cells for OHV-2 detection

For OHV-2 testing, DNA was extracted from the leukocyte fraction of blood for 42 animals caught in 2010. Initially, we used OHV-2-specific real-time PCR [20], but no samples were positive by this method. Therefore, we performed nested pan-herpesvirus consensus PCR [21] using a mix of degenerate and deoxyinosine-substituted primers (primary PCR: forward primers DFA and ILK, and reverse primer KG1; secondary PCR: forward primer TGV and reverse primer IYG) and HotStarTaq (Qiagen, UK). The thermal cycling conditions for both PCRs consisted of an initial activation step of 95 °C for 15 min, then 45 cycles of 30 s denaturation at 95 °C, 1 min of annealing at 46 °C and 1 min of strand extension at 72 °C, followed by a final extension step of 10 min at 72 °C. Amplicons were detected by electrophoresis in 1·5% agarose/TAE gel followed by staining with SYBR Safe DNA Gel Stain (Invitrogen, UK) and ultraviolet transillumination. Specific PCR products of 231 bp obtained from 21 of the samples were purified using ChargeSwitch PCR Clean-Up kit (Invitrogen) and sequenced in both directions using the primers TGV and IYG (Eurofins Genomics, UK). Sequences were obtained from 18 of these 21 samples. We assembled sequences using DNAStar (USA) and compared 178 bp sequences within primer sites to published data with a BLAST search (http://blast.ncbi.nlm.nih.gov/Blast.cgi).

### Statistical analysis of serological responses to *Leptospira* spp.

We analysed predictors of seropositivity (a binary variable reflecting whether an individual had an MAT titre that exceeded the positivity cut-off) using R v. 3.1.1 (https://cran.r-project.org/bin/windows/base/old/3.1.1/). First, we analysed predictors of seropositivity separately within each age group. We fitted a binomial error distribution and specified a logit link function. We included sex and years of birth and capture as fixed effects. Model variants were considered to improve fit if log-likelihood (LL) improved in likelihood ratio tests (LRTs), with the *χ*^2^ test statistic calculated as –2*(LL_model2_ – LL_model1_). The significance of changed LL between models was assessed using *P* values based on that *χ*^2^ statistic on 1 degree of freedom. Because of the small number of positives, we used exact logistic regression in the package elrm [22] to confirm and quantify predictors of seropositivity. Next, we analysed predictors of seropositivity in the 91 individuals tested both as yearlings and as adults, using linear mixed-effects models with a fixed effect of host sex and random effects of year of each capture (i.e. as yearling and as adult) and individual identity. Significance was determined using LRT, and results for the binary predictor of sex were confirmed with the non-parametric *ϕ* coefficient of association (to quantify the strength of association between sex and seropositivity) and Fisher's exact tests (to compute a test statistic and *P* value for that association).

## RESULTS

### No viral exposure detectable by serosurvey

We found no convincing evidence of exposure to viral pathogens by serosurvey. No samples from the 400 adult sheep contained detectable antibodies against BDV, PI3 V or orf virus (Table 2). We therefore did not test yearlings for these infections. We did detect antibodies against MVV in two adult sheep by ELISA. The samples exhibited optical densities (ODs) of 0·589 and 0·826, in relation to a negative cut-off of 0·231 and a MVV positive control of 0·776. However, 2/400 is within the false-positive rate for the test [23], and of the 350 samples from yearlings, none had detectable antibodies. At follow-up testing of the two putative positive adult samples by the more specific agar gel immuno-diffusion (AGID) assay [24], the positive results were not confirmed. These results of our major serosurvey are summarized in Table 2. Furthermore, the pilot serosurveys of 196 plasma samples collected in 1986 were negative for MVV, and the 50 samples tested in 2000 were negative for both MVV and BDV.

### Viral infection detectable by PCR of white blood cells

Initial analysis of white blood cell fractions from 42 sheep using OHV-2-specific primers in a real-time PCR assay [20] were negative. However, subsequent nested PCR using ‘pan-herpesvirus’ primers [21] revealed that 21 sheep were positive (Table 2); 18 contained OHV-2 DNA sequences, with results of insufficient quality for analysis in the other three samples. All 18 sequences were 100% identical to each other and 99–100% identical to published sequence for OHV-2 across the 178 bp region. Failure of the real-time PCR suggests low viraemia, consistent with previous suggestions that it may not be sensitive enough to detect OHV-2 DNA in sub-clinically infected animals [25].

### Bacterial exposure detectable by serosurvey

Of the 400 adults, four were weakly positive for *C. abortus*; in the yearlings, 28 came up weakly positive (at plasma dilutions 2–4/32). Similarly, for the separate sample set from the pilot serosurvey in 2000, 1/50 sheep was weakly positive (at 2/32) for this agent. Cross-reactivity between *C. abortus*, non-pathogenic enteric *C. pecorum* strains, and environmental bacteria such as *Acinetobacter* spp. can give rise to low false-positive results by this complement fixation test [26]. The low titres and the unexpected association with yearlings led us to suspect these were false positives. Indeed, follow-up tests on samples from our major serosurvey, using a *C. abortus*-specific indirect ELISA for antibodies against one of the polymorphic membrane proteins, POMP90 [27], showed that all 32 putative positive samples in our screening were actually negative (Table 2).

Of the 400 adults, six had detectable antibody against MAP; in 350 yearlings, two other animals were positive. Correcting for repeated sampling of 91 MAP seronegatives generated an overall seroprevalence estimate of 1·2% (Table 2). None of the 50 samples tested in the pilot serosurvey in 2000 were positive for antibody to MAP. No seropositive sheep in our major serosurvey died within a year of the observed seropositivity; indeed, some lived a further ⩾8 years. These observations would not be consistent with the course of clinical disease in MAP-infected domestic sheep [28], and the test is of imperfect specificity (98% [29]). However, the OD values for three of the eight positive samples exceeded the positive control, and several of the annual seroprevalences were well in excess of the expected false-positive rate (e.g. >10% seroprevalence in 2008; 7% in 2010). Unfortunately, we were not able to confirm our results using PCR or cultures because no suitably preserved faecal samples were available.

By contrast, we did obtain evidence of exposure of the sheep to leptospires. Of 400 adults, 28 had antibodies at a titre of ⩾100 at screening (i.e. detectable in a ⩾1:100 plasma dilution); 16 of the 350 yearling samples were positive by these criteria. The seropositives included one individual sampled as both a yearling and an adult. We thus found an overall individual-level (as opposed to sample-level) seroprevalence of 43/659, or 6·5%, across all years sampled (Table 2).

Upon more sensitive testing, many of the seropositives exhibited significant titres to *L. interrogans* sv. Hardjo or *L. borgpetersenii* sv. Hardjo: 15 adults and 13 yearlings exceeded the titre of 50 considered a firm positive for those serovars in the more sensitive follow-up assays [16]. When we determined the serovar(s) against which sheep exhibited maximum titres (following [30]), our data suggested that *L. borgpetersenii* sv. Hardjo may be most prevalent in this population. We found that 13 sheep exhibited titres of up to 200 against *L. interrogans* sv. Hardjo, but it was never the maximal titre for a sheep, while 34 sheep exhibited titres up to 1600 against *L. borgpetersenii* sv. Hardjo, and that serovar represented the maximum titre for 21 of the sheep. However, 40 sheep also exhibited positive titres up to 200 against *L. biflexa* sv. Patoc, and Patoc was the maximum or joint maximum titre (most often with *L. borgpetersenii* sv. Hardjo) in 31 sheep. Six sheep exhibited detectable titres against *L. interrogans* sv. Pomona (with titres up to 100), and Pomona was joint maximum only once (at a titre of 50 along with both *L. biflexa* sv. Patoc and *L. borgpetersenii* sv. Hardjo). Finally, only two sheep exhibited detectable titres against *L. borgpetersenii* sv. Ballum, and Ballum was never a maximum titre. In all, 34 samples had antibodies against one or more serovars; most singlets had antibodies against only *L. biflexa* sv. Patoc (six at titres of 100–200). Only one sample that originally screened positive subsequently had no detectable titres. The individual male who was positive both as yearling (in 2004) and adult (in 2006) bore antibodies against *L. biflexa* sv. Patoc on both occasions (titres of 100). It should be noted that bacterial isolates will be needed to identify the circulating serovar(s) definitively.

### Predictors of seropositivity for leptospires

**Fig. 2.**
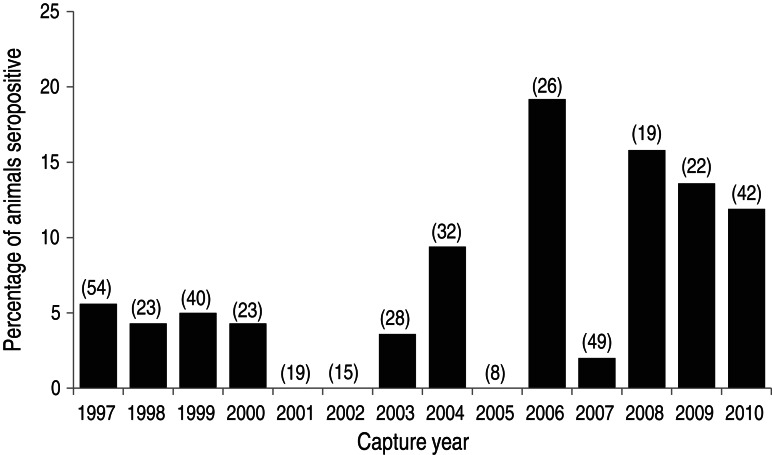
Annual seroprevalence of antibodies against *Leptospira interrogans* sv. Hardjo and *L. borgpetersenii* sv. Hardjo in 400 adult Soay sheep (aged 3–5 years) sampled during 1997–2010. A sample was considered seropositive at screening if it exhibited a titre of ⩾100 by microscopic agglutination test (MAT) against live bacteria. The number of adults sampled per year is shown in parentheses above each bar; no individual sheep was sampled more than once as an adult. For several years in the time series, very few samples were available due to population die-off the preceding winter. There was a significant increase in seroprevalence with time (see text).

We detected a significant temporal trend in seropositivity against leptospires in adult sheep. The full model suggested that, over the period 1997–2010, there was a significant increase in the proportion seropositive (*χ*_1_^2^ = 4·3, *P* = 0·039), and this finding was confirmed by exact logistic regression (odds ratio across years, e^*β*^ = 1·11, 95% confidence interval 1·01–1·25, *P* = 0·016; Fig. 2). Host sex was not significantly associated with seropositivity of adults (*χ*_1_^2^ = 1·9, *P* = 0·17), and we found no significant associations of year or sex with seropositivity of yearlings (all *χ*_1_^2^ < 0·05, *P* > 0·81).

**Fig. 3.**
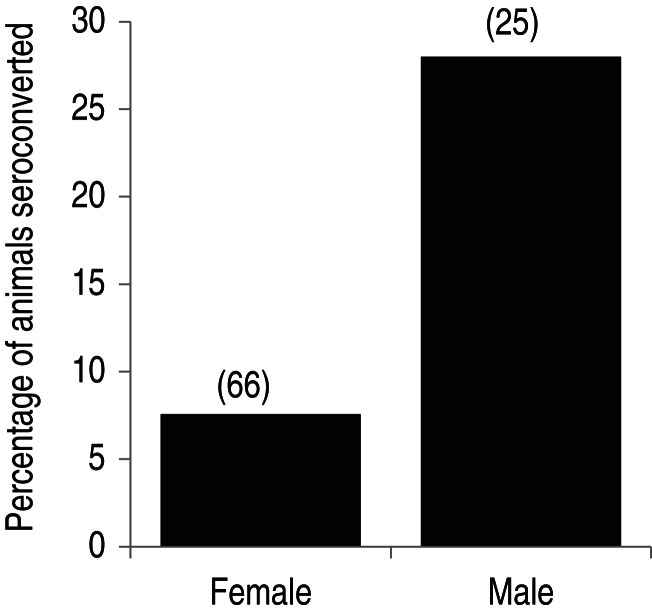
Seroconversion against leptospires between yearling and adult age groups, by sex, for 91 sheep sampled both as a yearling and an adult. A sample was considered seropositive if it exhibited a titre ⩾100 by microscopic agglutination test at screening. The number of individuals longitudinally sampled per sex is shown in parentheses above each bar. A significantly greater proportion of males seroconverted (see text).

Longitudinal analysis of the 91 individuals sampled both as yearlings and as adults suggested associations of seropositivity with sex and year of adult capture. One individual (a ram) was positive at both captures, and 12 individuals seroconverted between captures. Year of adult capture (*χ*_1_^2^ = 17, *P* < 0·0001) and a fixed effect of sex (*χ*_1_^2^ = 4·2, *P* = 0·040) significantly predicted seropositivity. A *ϕ* coefficient of association test, suited to assessment of association between binary variables in a small dataset and corrected for the number of individuals (91) rather than observations (182), confirmed that males were more likely than females to seroconvert between yearling and adult stages (*ϕ* = 0·27, for *χ*_1_^2^ = 6·6 and Fisher's exact *P* = 0·016; Fig. 3). There was no difference between sexes in the interval between samples.

## DISCUSSION

We found evidence of exposure of the Soay sheep on Hirta only to the persistent and/or non-immunizing infections from this list, and to just a subset of those (Table 2). While serological testing cannot prove the absence of a pathogen beyond doubt, consistently negative results provide good evidence for freedom from infection. For example, our results are adequate to conclude that the population is free from MVV at the expected minimum prevalence of 0·2% and at the 0·9965 confidence level; this freedom-from-disease analysis assumes test sensitivity of 0·90 and specificity of 0·99 [31]. Furthermore, for readily transmitted and strongly immunizing viruses such as BDV, PI3 V and orf virus, given the sensitivity and specificity of these tests [32, 33] and the high transmissibility of these infections (e.g. [34]), the consistently negative results over a decade of sampling are suggestive of freedom from infection for these three viral agents as well. The apparent absence of these infections from the Soay sheep of St Kilda contrasts with a Scotland-wide, 2007 serosurvey for BDV, PI3 V and MVV in 125 sheep flocks, including tests of 27 ewes from each of 50 flocks (and a total of 1350 ewes) in the Highlands and Islands. That study revealed seroprevalences of up to 20% for BDV, nearly 100% for PI3 V, and up to 10% for MVV [13]. The total sample size of 659 Soay sheep in our major serosurvey (with up to 85 individuals from the ‘flock’ tested per year) was powered to detect such seroprevalences. We therefore suggest that more exhaustive sampling would probably not have yielded seropositives in the years sampled.

By contrast with the acute immunizing infections discussed above, pathogens such as *C. abortus* cause chronic infections and can be maintained despite low incidence and prevalence. Nonetheless, we found no convincing evidence to suggest exposure to this pathogen in the sampled Soay sheep. MAP also causes persistent infection, and we found low seroprevalence by ELISA (<2% across years, although seroprevalence was >10% in 2008 and >7% in 2010). However, we were not able to undertake confirmatory culture of faeces. The presence of MAP among Soay sheep on St Kilda therefore remains to be determined.

We did find evidence of exposure to other pathogens associated with chronicity and/or subclinical carriage in domesticated Scottish sheep. For example, we detected OHV-2 DNA within circulating leukocytes of 50% of sheep tested. Further sampling and sequence analysis that targets polymorphic regions of the OHV-2 genome would be necessary to investigate long-term prevalence and genetic origins of herpes infecting St Kilda Soay sheep. It could be of interest to compare their herpes to herpes in related/ancestral sheep breeds.

We also found that, in some years, seropositivity against leptospires approached 20% (Fig. 2), which raises questions about the identity of the serovar(s) and variation in the frequency and route(s) of transmission. Most *Leptospira* spp. can circulate among various host species. However, leptospires cause chronic, subclinical infections only in ‘maintenance’ or ‘reservoir’ hosts. For example, Hardjo serovars circulate among ruminants, often with cattle as the reservoir [15]. Ballum, by contrast, infects sheep but uses rodents as reservoirs [16]. When reservoir hosts are removed from a community, transmission to non-reservoir hosts should fall to zero. Given that cattle have been absent from Hirta since 1930 [6], our results suggest that an ovine population might indeed be capable of maintaining transmission for the *L. borgpetersenii* Hardjo serovar [35]. However, there are limitations to leptospire-specific serology. For example, the sensitivity of MAT can be as low as 40%, even when clinical signs and urinary excretion of bacteria are observed [36]. Definitive serovar identification and prevalence estimation on Hirta would thus require isolation of bacteria from urine of sheep and the endemic mouse species (*Apodemus sylvaticus hirtensis* [37]). It should be noted that government and other officials make vigorous efforts to monitor for rats, especially after any shipwrecks near Hirta; all lines of evidence to date suggest that the island remains rat free.

Conservation or management concerns become paramount when immunologically naive island inhabitants are exposed to mainland parasites (e.g. canine distemper virus purportedly in island foxes [38] or ocelots [5]). This study suggests that the sheep on St Kilda experience a wider variety of chronic, endemically transmitted infections than previously appreciated [8, 39] and raises many new questions about both inter-individual and inter-annual variation in the dynamics of infection. For example, resistance against nematodes is associated with improved survival of the Soay sheep [40], and effects of nematodes on host condition appear independent of coccidian co-infection [39]. But how might herpes or pathogenic *Leptospira* co-infection affect survival, fecundity or immunosenescence of Soay sheep? And would any of the cross-reactive antibody types detected in the sheep (e.g. autoantibodies, natural antibodies and/or IgM [40]) confer protection against these infections? Furthermore, how might the ~10-day variation in median birth date [6] and other demographic or environmental sources of inter-annual variation affect transmission dynamics? We look forward to discovering answers to these questions.

Empirical tests have borne out predictions of island biogeography [1] in several host–parasite systems (e.g. [5]). Here, we report that Soay sheep of St Kilda also harbour depauperate pathogen communities. Our findings accord with the ecological expectation that acute immunizing infections could not persist [3] on Hirta. The power of this system is that, thanks to the long-term study of the sheep [6], we can now begin to investigate the demographic consequences to the sheep of harbouring the few pathogens and parasites with life histories that enable persistence on their windswept island.
